# Cooperation in Return-to-work Interventions for Common Mental Disorders: An Ideal Theory Analysis of Actors, Goals, and Ethical Obstacles

**DOI:** 10.1007/s10728-024-00491-1

**Published:** 2024-09-17

**Authors:** Thomas Hartvigsson, Lars Sandman, Gunnar Bergström, Elisabeth Björk Brämberg

**Affiliations:** 1 Institute of Environmental Medicine, Unit of Intervention and Implementation Research for Worker Health, Karolinska Institutet, Stockholm, Sweden; 2 Department of Philosophy, Linguistics and Theory of Science, University of Gothenburg, Gothenburg, Sweden; 3 National Centre for Priorities in Health, Division of Society and Health, Department of Health, Medicine and Caring Sciences, Linköping University, Linköping, Sweden; 4 Department of Occupational Health Science and Psychology, Faculty of Health and Occupational Studies, University of Gävle, Gävle, Sweden; 5 Institute of Medicine, School of Public Health and Community Medicine, University of Gothenburg, Gothenburg, Sweden; 6 Centre for Ethics, Law and Mental Health, University of Gothenburg, Gothenburg, Sweden

**Keywords:** Ethical frameworks, Return-to-work, Sick leave, Common mental disorders, Cooperation, Institutional ethics

## Abstract

The rise in the number of people on sick leave for common mental disorders is a growing concern, both from a societal and individual perspective. One common suggestion to improve the return-to-work process is increased cooperation between the relevant parties, including at least the employer, the social insurance agency and health care. This suggestion is often made on the presumption that all parties share the common goal of reintegrating the patient-employee back into the workplace. In this paper we investigate this presumption by mapping out the ethical frameworks of these three key actors in any return-to-work process. We show that although the goals of these actors often, and to a large extent, overlap there are potential differences and tensions between their respective goals. Further, we emphasise that there may be other limitations to an actor’s participation in the process. In particular the health care system is required to respect patient autonomy and confidentiality. There is also an inherent tension in the dual roles of health care professionals as therapists and expert witnesses in work ability assessment. In conclusion, there are potential tensions between the key actors in the return-to-work process. These tensions need to be addressed in order to enable an increased cooperation between actors and to facilitate the development of a feasible plan of action for all parties, including the employee.

## Introduction

The rise in the number of people on sick leave for common mental disorders (CMDs)^1^ – a term incorporating depression, anxiety and stress-related disorders – is a growing concern from a societal and individual perspective. In OECD countries almost one in five suffers from a mental health condition, of which CMDs constitute the major part [5]. The total costs of mental ill-health are estimated to be more than 4% of GDP. For the individual, in addition to the direct suffering involved, CMDs can lead to sick leave or unemployment, which in turn is associated with a worse economic situation, social isolation, deteriorating mental health, and shorter life [6, 7]. Hence, having people with CMDs on sick leave (re)enter the work force is a key priority.

To this end, cooperation between the relevant actors (i.e., the employer, health care organisation and the social insurance agency) is a commonly suggested measure for improving the return-to-work process [8–11]. This suggestion is underpinned by the presumption that all actors share the goal of reintegrating the employee at work. However, the return-to-work process is complex: it involves several different actors, each with their own ethical framework. In this paper we question this presumption.

The actors’ ethical frameworks consist of goals and ethical principles. These goals and principles can come into conflict, fall short of the reintegration goal, or constrain the agent’s participation in the process. Adherence to the ethical framework is enacted through laws, policies and individual actions. The ethical framework acts as a justificational base in institutional decision-making. Merely having more than one actor opens up for problems associated with cooperation, communication, and coordination. Tensions between the different actors can go beyond pragmatic issues, and reflect underlying conflicts between the ethical frameworks. By making an ideal theory type of analysis, in which we assume adherence by the institutional agents to their respective ethical frameworks (cf. [12]), we lay bare inherent ethical tensions and potential conflicts between the actors. Hence, the aim of this paper is to map out the ethical landscape: the different institutional ethical frameworks and their interrelations, in the return-to-work process for people on sick leave due to CMDs. Through our analysis we show that that the presumption of a common goal does not hold: it might be true in some or even most cases but will not be true in all.

We limit our analysis to a minimal set of actors: the health care organisation, the social insurance agency, and the employer. This makes the analysis manageable but sufficiently comprehensive. Other relevant actors (with partly overlapping ethical frameworks) are for example occupational health services and employment agencies. The three chosen actors also coincide with three of the policy areas highlighted by the OECD [5] in tackling the mental health problem: the health care system, the welfare system and work policy.

We will mainly use examples from the Swedish context to illustrate how the ethical frameworks can manifest. Currently, in Sweden, there is an ongoing process of assessment and improvement of the cooperation between the actors in the return-to-work process, with an emphasis on the role of the social insurance agency [13, 14]. This has led to some changes in law [15] and practice. This process is motivated by mental disorder becoming the most common reason for sick leave: annually, about 3.4% of the workforce receives sickness benefits for a mental disorder and at least 80% of the men and 90% of the women on sick leave due to psychiatric disorders are so due to CMDs [16]. Our aim here is to inform this continued process as well as the development and implementation of targeted interventions based on cooperation between the three actors [4]. Even though Sweden provides a unique example we hope that other countries can benefit from our analysis. The institutions, laws and policies are particular to a country and rooted in its history and culture but the underlying ethical values and principles are shared across borders. This paper may be instructive with regards to how these ethical principles and values can be enacted and balanced. It thus has relevance for all countries with similar institutions.

The three actors are introduced in order of increasing complexity in their respective ethical frameworks, beginning with the ethical framework of the employer, followed by the social security agency and finally the health care organisation. We explore the relationship between these ethical frameworks systematically. Each section follows the same format: we present the goals of the institution followed by relevant constraints. After having presented the goals of an institution we analyse how these goals relate to the goals of the institutions previously presented, aiming to outline all the possible conceptual and causal relationships between the institutions’ goals. We also note how constraints limit the pursuit of previously presented goals.

## The Employer

The overarching goal for the employer is to produce goods and services. Additionally, the employer has a legal responsibility for the work environment. In Sweden, this includes extensive preventive responsibilities [17] and an obligation to facilitate and participate in the return-to-work process [15]. Irrespective of what caused an employee’s CMD, active participation from the employer is usually necessary for a successful reintegration back into the workplace. These latter obligations may be both conducive to, and limit, productivity.

### Productivity

There exists a plethora of employers with different aims, modes of organisation and ethical ideals. However, regardless of what the precise goals of an employer are, we propose that these can be subsumed under the heading of productivity. It has been argued that the sole goal for companies is to make a profit [18]. This view has been widely criticised for taking a too narrow approach to corporate social responsibility (see e.g. [19, 20]). However, even the critics agree that profit is one legitimate goal. Not all employers are for-profit corporations. Non-profit organisations and public agencies may have other goals. Yet, arguably, they also want to use their resources efficiently in the pursuit of their goals. Stipulating productivity as an overarching goal for employers encompasses a range of different goals. Limiting our analysis to this goal also keeps our analysis manageable, while allowing us to tease out the relevant tensions. It is also reflective of an agenda stressed by employers in the return-to-work process [21, 22],

### Productivity and Return-to-work

When an employee becomes ill, the employer will suffer a productivity loss. The tasks not done by the sick employee will not be done, be done by an additional person, or be spread out among the absent person’s colleagues. That is, either, the tasks will not be done, or they will be done at an increased cost, or they will be done at an increased cost and increased risk for further sick-leave due to the heavier workload imposed on the other employees, which in turn gives rise to the same dilemma.

Concerning the return-to-work process, four different scenarios are possible from the employer’s perspective when an employee becomes ill and the outcome of the rehabilitation process is uncertain.

1. The employee returns to work without any changes.
2. The employee returns to work with adjustments.
3. The employee returns to a new position for the employer.
4. The employee does not return to work.

Which of these scenarios the employer desires will differ between cases. For an employee who returns to work with no changes there will be no extra direct costs for the employer. However, if the performance of the employee is reduced then they will produce less for the same cost. If the reduction is due to remaining ill-health this will be a case of presenteeism [23]. If work was a contributing cause of the CMD motivating sick-leave, then making no changes also means that there is a push for renewed sick-leave. If the employee returns with adjustments, it could mean either a reduction in productivity or more direct costs for the employer. The third scenario means that a replacement needs to be found, as well as a new position for the employee. How much costs this incurs will vary depending on the position as well as available labour. Further, not all employers will be able to find a new position for the employee. The fourth scenario, to let the employee go and have someone else do their work will at least for an initial period of time incur a lowered productivity while the replacement learns the job. The replacement is, from the perspective of the employer, hopefully more productive than the former employee. In order to speed up this process, the employer can participate in helping the employee find a new job. From a strict productivity perspective, different scenarios will be desirable in different cases. If replacing the employee on sick leave would increase productivity, then the employer does not have an interest in participating in the return-to-work process.

### Employer Responsibility and Work-environment

In Sweden, employers are obliged to adhering to the Work Environment Act, e.g., to provide a safe and health-promoting work environment. This is motivated by beliefs that a good work environment is conducive to worker productivity [24] and by a genuine care for the employees. Yet this does not preclude that there are situations in which there is a conflict between promoting a good work environment for the individual employee and the productivity goal. This conflict may be exaggerated by an emphasis on short-term productivity goals, while ignoring long-term benefits of a good work environment that includes low numbers of sick leaves and low staff turnover.

Despite this, there might still be instances of genuine conflict between productivity and a healthy work environment, in particular when employees can easily be replaced. To what extent this is the case depends on transaction costs, such as how long it takes to learn the to do the job, as well as the supply of potential workers. Further, legislation pertaining to work environment and sanctions for violating these rules can change the balance for how to prioritise when these two concerns conflict.

The employer’s responsibility in the return-to-work process is most clearly in conflict with the productivity goal when the employer does not want to keep the employee. A strict focus on productivity implies that low performing employees should be replaced. The employer has considerable discretion in choosing to what degree they participate in the return-to-work process. They usually have the ultimate say in whether any work-place adjustments are possible or if there are other positions available.

## The Social Insurance Agency

The primary purpose of social insurance is to pay just compensation to people who due to illness are unable to work. People only have a right to compensation to the degree to which they are unable to work. In the case that some work ability remains, the employee should, if possible, work to the corresponding degree. Due to the benefits of employment from a societal as well as individual perspective, restoring or promoting work ability is an important goal. Work ability is thus a central concept for the three goals of the overall social insurance scheme:

1. to assess and pay compensation for reduced work ability due to illness.
2. to support restoration or promotion of work ability.
3. to support the employee to work to the degree of their work ability.

The Swedish social insurance agency (Försäkringskassan) has the responsibility to assess the work ability of the employee, decide if they are entitled to sickness benefits and administer the sickness benefits. The agency does this based on medical assessments from health care professionals and information from the employee and the employer. Further, the Swedish social insurance agency is responsible for coordinating the relevant actors (e.g., health care, employer, occupational health services) and their interventions aimed at promoting work ability. They can also compensate someone who takes part in a rehabilitation program. Refusal to participate in rehabilitation is grounds for withholding sickness benefit payment [25]. The Swedish social insurance agency thus is tasked with assessing work ability and paying compensation, in addition to supporting the promotion of work ability. Refusing to work to the degree that one can is grounds for dismissal or ineligibility for other benefits. Overall, the Swedish social security scheme encompasses the three goals.

### Work Ability

The notion of work ability is central to the ethical goals of the social insurance agency, yet no standard definition exists (see [26] for an overview). Regardless, there are two different conceptions of the notion; specific and general. Specific work ability refers to the abilities and skills necessary to do a specific job and general work ability refers to the ability to do some kind of work. In Swedish law this means assessing a person’s ability against the hypothetical construct of a “normally occurring job” whereas other legislation might use other benchmarks, such as actually occurring jobs [27]. In this paper we predominantly focus on specific work ability since we are interested in the employee’s ability to return to their previous job or some other specific job that the employer can provide.

Health is a constitutive part of work ability, but the relationship between the two notions is complex. Work ability requires that a person has the ability to do either a specific job or could fulfil the requirements of some more general job construct. This requires some degree of health. What degree of health depends on the nature of the particular job. Furthermore, some people may be able to compensate for a deficiency in health by other abilities, meaning that some people may require a higher degree of health to have work ability than others. Finally, very few jobs require that a person is in full or perfect health to have work ability [27–29].

In addition to health, a person also needs a certain amount of training and skills. In the developed world some degree of basic education is necessary for almost any job, and therefore for general work ability, whereas specific jobs might require a great deal more education [27–29]. It has further been suggested that work ability also includes virtues, motivation [27–30] and other qualifications [28].

### Goal 1. Just Compensation

A work ability assessment is necessary for determining the degree of illness-reduced work ability. That sickness benefit exists and can be relied upon is beneficial for the mental health of the employee. Being denied sickness benefit payments, in particular after having relied on this, is a source of great distress [31].

Work ability assessment can be more or less rigorous and demanding for the person assessed. Active participation in the process can be taxing. Going to meetings and assessments, as well as dealing with the bureaucracy takes time and effort. Additionally, uncertainty about whether the sickness benefit claim will be accepted likely contributes to distress. That the time and energy of the claimant go towards fulfilling the requirements of the assessment process means that this time cannot be put into productive labour, and hence reduces a person’s work ability. In particular, work ability may be hampered if the outcome of the process is uncertain and therefore stressful.

When developing work ability assessment practices, further considerations are relevant in addition to the three goals related to work ability. The risk for benefit fraud and costs associated with the assessment process are also important concerns.

### Goal 2. Support Restoration or Promotion of Work Ability

The social insurance agency does not, at least in the Swedish case, directly engage in promoting work ability. The task of the agency is to facilitate and support the other agents who are directly engaged in promoting work ability. As a multidimensional and relational construct, work ability can be promoted in several different ways, where health promotion is one possibility. It can further be promoted through training and skill development, or adjusting work assignments by removing certain tasks, changing the circumstances of doing them or adjusting the nature of them.

The social insurance agency’s aim is to have a person’s work ability restored so that they can be reintegrated back into their workplace. As noted in the previous section, employers may not be interested in reintegrating an employee back into the workplace. In this case the employer does not share the same goal as the social insurance agency.

Further, even in the case the employer wants to reintegrate the employee, and hence has an interest in promoting their work ability, they may have a different idea compared to the social insurance agency of when this has been satisfactorily accomplished (and what it should reasonably cost the employer). From the perspective of the social insurance agency, it is enough if the employee can do their job to a satisfactory or adequate degree, whereas an employer wants their employee to be maximally productive. So even if both parties express an interest in promoting an employee’s work ability, they may have different ideas about how, and when, this goal has been reached.

### Goal 3. Utilising Work Ability

If the employee on sick leave has some degree of work ability they should, if possible, work to that degree. In the Swedish context, refusal to do so is grounds for dismissal or ineligibility of benefits. Sick insurance only compensates a person to the extent that their work ability is compromised by illness.

Having a person on sick-leave return partially to work is a common and important step in the return-to-work process. This also allows for work-based interventions aimed at promoting the employee’s work ability [4]. A partial return to work may thus promote both the goal of utilising the degree of work ability a person has as well as promoting their work ability.

However, having an employee return to work may also work in the opposite direction. When work was an important factor in making the employee ill this is a salient risk. Risk factors connected to the employee include previous sick absence and higher symptom scores whereas positive work expectations, high return to work- and self-efficacy scores and high workability index increases the chance of a successful return-work process [32]. Simultaneously, some jobs, particularly in the public sectors, carries increased risk for CMDs [16] and non-ideal employers may fail to remove well-known risk factors [33–35]. Further, although a partial return to work does not reduce work ability, it may still stifle its improvement and thus limit it to a reduced level. For a person who has some degree of work ability, utilising this by having the person partially return to work can affect their work ability in three ways: improve it, keep it stable, or make it worse.

Although all parties involved in the process may agree that both the goals of promoting and utilising work ability are desirable, the emphasis put on each respective goal and on how to balance the risks associated with return to work and continued sick leave may differ. Both (partial) return to work and continued sick leave has its risks and potential benefits and these need to be weighed against each other. Employers and employees may prioritise return to work and avoiding future illness differently [8].

## The Health Care System

The health care system has two primary functions in the return-to-work process. Firstly, it diagnoses and treats the illness underlying the reduction in work ability. Secondly, it takes part in the forensic process by providing expert testimony about the nature and impact of illness to the social insurance agency. Compared to employers and the social insurance agency, the health care system has a well-developed ethical framework that is firmly rooted in the professions.

In this context, the main goal of the health care system is to promote health. This goal is clearly articulated in a key paragraph of the Swedish Health Care Act, which states that the goal of health care is a good health and equal access to health care for the entire population [36, Chap. 3. 1§]. We will explore this goal and discuss it in relation to the goals of the two other institutions. Further, there are limits to pursuing the goal of health within health care ethics underpinned by considerations of distributive justice and health care prioritization, as well as autonomy and confidentiality. We will also explore the relevant implications of these considerations. Finally, we explore the tensions between the two functions of the health care system: the therapeutic and the forensic.

### Promoting Health

There are different approaches to understanding health. One such approach is to understand health in terms of well-being. Famously, WHO defines health as “a state of complete physical, mental and social well-being and not merely the absence of disease or infirmity” [37]. However, this definition suffers from a number of identified problems. First of all, if the goal of the health care institution is to promote health, then according to this definition there is, practically, no end to health care professionals’ obligations [cf [38, 39]. Secondly, there are also some conceptual issues with this definition. Defining health in terms of well-being increases rather than settles confusion since the concept of well-being is at least as contested as the concept of health [40]. Furthermore, it seems conceptually possible that a person’s well-being can be improved despite that person being in full health. The WHO definition does not allow this possibility [38]. However, we do acknowledge that the WHO definition importantly signals that the value of health is not only about absence of disease but its broader role in enabling and promoting a good or flourishing life.

Another approach to understanding health is to understand health as a set of functions. It should be noted that in practice the WHO does not adhere to its own definition of health. Both the International Statistical Classification of Diseases and Related Health Problem (ICD-11) and the International Classification of Functioning, Disability and Health (ICF) understands health in terms of functioning.

Several attempts have been made to try to define health and pinpoint which functions that constitute health. Some suggestions in the literature are the ability to satisfy basic needs, “to do ordinary things”, to run our daily affairs, to make ourselves minimally happy [41], or species-normal functioning [42]. In addition other dimensions such as absence of certain kinds of suffering may be added as well [43]. For this paper we understand health to mean a set of basic functions or abilities. For present purposes we do not need to accept a particular theory of health, instead we use basic functioning as a stand in for the correct theory of health.

Common mental disorders impact basic functioning in many ways. Lack of energy means that the patient has a hard time initiating and execute everyday tasks. The patient may no longer be interested in activities that they used to enjoy. Impaired cognition may take the form of decreased ability to concentrate and memory problems. Mental distress and suffering due to sadness, irritability and anxiety may further impair the patient’s ability to function and their ability to work as well as their ability to manage social relations and their lives. These examples bring us back to the focus of the WHO definition: the focus on flourishing for which health plays a pivotal role.

The key feature, for our argument, of plausible health theories is that there is an upper limit to the degree of functioning beyond which further improvement does not count as improvement in health. For example, some degree of memory is necessary for mental health, however, some people possess tremendous capacities for remembering things, but this does not mean that they have greater mental health than someone with a more ordinary memory. Some jobs might require abilities that go beyond what is necessary to be healthy. Even so, employers can be expected to want their employees to function above the minimum threshold required for the job if this means a higher employee productivity. If the requirements of a particular job are higher than the threshold for health, then the health care system does not have fully restored work ability as a goal.

Furthermore, being healthy requires a number of different functions, which means that it might be necessary to make trade-offs between different functions. If health consists of additional dimensions, such as suffering, more potential trade-off situations exist. Not all aspects of health will necessarily be constitutive of work ability. Axén and colleagues [44] found that reduction of symptoms and increase in work ability could be independent outcomes of treatment. This shows that, when different treatment options are considered, a decision sometimes needs to be made about which aspects of health to promote. There is nothing inherent in the aim to promote health generally that supports prioritizing the functions underpinning work ability rather than other aspects of health.

### Concerns of Distributive Justice and Health Care

Health care has limited resources. There will always be greater need for health care than resources to provide it. Hence, it is necessary to prioritise among the resources available. The key paragraph of the Swedish Health Care Act mentioned previously declares that health care should be delivered with respect for all people’s equal worth and respect for human dignity and that priority should be given to those with the greatest need. This part of the paragraph has been interpreted in the Swedish Ethical Platform for Health Care Prioritisation [45]. The principles in the ethical platform are not unique as such, even though their strict ordering and explication offer an example of a very clearly articulated set of national guidelines on health care prioritisation.

## The Ethical Platform for Health Care Prioritisation

The ethical priority platform consists of three principles, with a rank order. The principles are:

1. *The human dignity principle.* Explicitly this principle claims that priority setting should not be based on personal characteristics or standing in society. It can be viewed as a formal equality principle or anti-discrimination principle, explicitly stating that social function or standing, economic standing, chronological age or previous lifestyle should not be taken into account.
2. *The needs and solidarity principle.* This principle states that those with the greatest need should be given priority where need is understood in the dimensions of suffering, medical prognosis, functional impairment, and existential needs due to the condition. The principle also requires interventions to have a patient benefit.
3. *The cost-effectiveness principle.* This principle claims that there should be a reasonable balance between the cost and effects of interventions.

In practice, the rank order of the principles is understood in terms of accepting a worse ratio between cost and effect for more severe conditions. In other words, health care should spend relatively more resources on more dire conditions or needs. Mental disorders can no doubt be serious and rank very highly on these principles. However, for example in the case of CMDs, when work ability is only partially reduced this will get a lower priority, with implications for how much resources should be spent and to what extent patients should get access to care.

The cost-effectiveness principle can limit health care resources for a person in a return-to-work process. Even if a person with a CMD gets a relatively high priority based on the severity of their condition, getting the resources required to fully restore health may not be cost-effective enough. The patient will then have to settle for care that only partially, but not fully, restores health.

### Promoting Health and Work Ability

Not working can be detrimental to a person’s mental health [6, 7]. Restoring work ability and having the person return to work, if only partially, is therefore in many instances key to improving or preventing further deterioration of a person’s mental health [46, 47]. Health, at least at a very basic level, is a fundamental precondition for work ability and promoting health is one way of promoting work ability. Therefore, the aims of promoting health and work ability are expected to coincide in most cases.

The notion of ability is central to both health and work ability. It was observed that, at least for some jobs, abilities beyond those considered as full health might be required for full work ability. It is then not within the aims of the health care system to restore that degree of work ability, but merely as much as is encompassed by full health. Health care priority considerations may further lower the aims of the health care system vis-à-vis work ability, as the needs of a patient with reduced work ability may not be enough to warrant treatment or the cost-effectiveness of available treatments might not be good enough. Health care’s goal of promoting health may thus be reached before full work ability has been restored.

In addition to disparities between when institutional agents are satisfied with the promotion of a person’s work ability, the goal of promoting health can come into conflict with the previously mentioned goals. Firstly, it was observed that some treatments may reduce symptoms but not increase work ability and vice versa. Secondly, just as the aim of promoting work ability can come into conflict with the goals of just compensation and utilising existing work ability, so can the goal of promoting health come into conflict with these as well.

### Autonomy

One of the key principles in modern medical ethics is respect for patient autonomy [48]. The core of this principle is to have the patient make decisions regarding their own care. Traditionally this requires that the patient gives their informed consent before a medical treatment can be given. Within a person-centred approach where shared decision-making is practiced greater patient involvement and incorporation of the patient’s preferences in the medical decision-making allows for a more flexible approach to patient autonomy than merely allowing the patient to refuse or accept care [49, 50].

In the standard case, before treatment can begin, the patient must give their informed consent to the treatment in question. A patient’s consent is considered valid if they have been adequately informed about the nature and potential risks and benefits of the proposed treatment, if they have mental capacity to make a decision based on that information, and if they are not subject to undue influence [48]. We will assume that the health care professionals are doing their jobs and are informing the patient properly. Further, we will assume that the patient is competent. Although mental disorder can undermine mental capacity, CMDs are comparatively mild, which makes this less likely^2^. Further, if the CMD undermines the patient’s mental capacity, restoring it is probably the correct priority^3^.

For different reasons the patient may not want to pursue the option that best improves their health or work ability. The patient may refuse consent to any efficient treatment, or the patient may, within a shared decision-making process, opt for a suboptimal treatment. Accepting this is to respect the patient’s autonomy but is at odds with the goal of promoting health and the goal of promoting work ability.

There may be external pressure on the patient to make certain decisions regarding treatment. For example, from the patient’s family, friends and employer. These influences can be strong and push the patient in a direction that may not reflect their own will and risk becoming undue. In particular the power wielded by the employer in this context merits further analysis as they are a central gatekeeper for the patient’s return to work [21].

In ordinary circumstances the pressure from the social insurance agency, to accept care that restores work ability, is likely the strongest as the patient’s claim to sick insurance depends on them accepting such care. Assuming that such conditions on behalf of the social insurance agency are just this does not constitute undue influence on the patient’s decision but raises an ethical question for the health care professionals if and how to communicate that a patient does not want the treatment that best restores the work ability that provides a segue to the next two sections as this problem is central to them.

### Health Care Professionals as Experts in Forensic Procedures

Health care professionals have an important role as expert witnesses in the assessment of work ability and hence have a key role in the process of administering sickness benefit. It is common that the treating physician also holds the forensic role of expert witness by providing information to the social insurance agency about the nature and impact of the illness on work ability. It is widely recognised that there is a conflict between the role as treating physician and expert witness in a sickness benefit process [25, 51–53].

As opposed to health care practice where the aim is promoting patient health, the aim of expert testimony is truth [54]. The medical expertise possessed by health care professionals can be used to assess the nature of an illness and its impact on a person’s health. These kinds of assessments are essential to many legal processes where that information is essential for determining whether and to what extent a legal rule applies. However, the translation between the language of the medical expertise and the language of the law is not always easily recognisable. For example, even though a health care professional can appreciate the nature of a depression and how that affects a person’s life with a professional degree of precision, it is then a further question of if and to what extent this can be translated into the language of the social insurance agency. In Sweden that means translating the reductions of work ability to 25% intervals [21]. Making work ability assessments is also not part of the training required for having a treating role, and health care professionals, although expected to make such assessments, may lack adequate training for this [55–57].

The truth-seeking goal can come into conflict with the therapeutic role in more subtle ways. Firstly, the therapeutic relationship is based on trust and empathy, whereas the forensic role is characterised by scepticism and a desire to, if possible, control the veracity of the patient’s statements [58–62]. The search for truth may call for a different approach to how questions are asked and as such change the dynamics of the patient consultation [61]. There might also be reasons to place emphasis on different things in the two roles. One central aspect of a therapeutic relationship is to convey comfort and hope, as long as this is reasonable. A more frank statement of the prognosis as might be appropriate in the legal proceeding can send the opposite signal to the patient [61]. Finally, part of the clinical consultation is to form a therapeutic alliance. For this the patient needs to feel that the health care professional is on their side. Accepting a patient’s express desire for sick leave is one way of doing this and physicians have a hard time denying such requests [63, 64].

### Confidentiality

Confidentiality is a cornerstone of the therapeutic relationship between health care professionals and patients [48, 65]. During the consultation sensitive information about the patient will be discussed, primarily information with clinical relevance but other kinds of private information may also be shared. Sharing this information with a third party without the patient’s consent is a breach of confidentiality. Yet sharing such information with other actors such as occupational health services or the employer may be crucial to a successful return-to-work process. If the patient does not consent to the sharing of this information then they are hampering efforts aimed at promoting their health or work ability.

Patient confidentiality is at obvious odds with the forensic role of health care professionals [62]. The aim of providing the social insurance agency with accurate information involves sharing sensitive information with them. If a negative decision for the patient is expected to worsen their health, there is a reason grounded in the ethics of the therapeutic role not to share that information. Further, the patient is likely to oppose health care professionals sharing information if they think that this information will affect the social insurance agency’s decision negatively. In fact, the fear of having health care professionals share such information incentivises the patient not to share it with the health care professionals in the first place. This in turn means that clinically relevant information may be withheld from the health care professionals and thus threaten the quality of care [58, 62].

## Concluding Discussion

The rise in the number of people on sick leave for CMDs is a growing concern, both from a societal and individual perspective. One common suggestion to improve the return-to-work process is to suggest increasing cooperation between the relevant parties. This suggestion is often made on the presumption that all parties share the common goal of reintegrating the patient-employee back into the workplace.

In this paper we have shown that this presumption does not always hold. The employer, if only guided by a goal of productivity, may not be interested in reintegrating an employee at the workplace. Further, the employer wants employees who are maximally productive, whereas the social insurance agency is satisfied when the employee can meet the minimum requirements of their job. The goal of health care is to promote health within the limits imposed by health care prioritization. Health care professionals may thus be content with their contribution even if work ability is not fully restored. We have further observed that other goals such as assessment of work ability and pursuit of just compensation, as well as utilising remaining work ability can counteract the promotion of work ability and health. Finally, health care ethics contain three constraints pertaining to the participation of health care in the process: respect for autonomy, confidentiality, and the therapeutic role. This together with inconclusive evidence for how the cooperation between the actors should be enacted to increase workplace reintegration in the case of CMDs [44, 66] highlights the need to address these challenges when proposing increased cooperation.

This paper has used ideal theory approach, yet we know that many actors fail to live up to the ideal. Some jobs carry a higher risk of employees developing CMDs. In particular, jobs found in public administration, social services, health care and education carry an increased risk for CMDs [16]. These areas of work are essential to the welfare state and often involve care and responsibility for vulnerable people. Traditionally these jobs are found in the public sector. Further, there is also an increased risk for people in managerial positions and occupations which require a university degree. There might be something intrinsic in these jobs which carries an increased risk, but they may also be characterised by a number of well-known work-related risk factors. These risk factors include high workload, low reward and support, and job insecurity [33–35]. These factors are within the employer’s control and a failure at the preventive stage does not inspire confidence that employers will take appropriate responsibility in the rehabilitative phase. Examining how the different actors fall short of their ethical obligations is an important task of empirical research and this paper contains a map of what to investigate. Then there is the additional, partially ethical, question of how to deal with such shortcomings.

This paper also raises further ethical questions. We have touched upon the responsibility of the patient-employee. What are their obligations in the process and in what ways may they fall short of these? It is unlikely that all people on sick leave have an interest in returning to their jobs, yet this is presupposed in the process. Further, both work and private life can contribute to CMDs, which raises the question of what obligations a person has to manage their private life in a way conducive to maintaining and restoring work ability. This question is also connected to ongoing debates about health care priorities and patient responsibility for their health [67].

An additional question concerns health care prioritization in relation to the return-to-work process. According to the Swedish platform for health care prioritisation, it violates the human dignity principle to consider the economic contribution of a patient when making a priority decision [45]. As seen above, both from the employer’s and the social security agency’s perspective, it is essential to get the person back into productive labour. An absent employee incurs both direct and indirect costs to society in the form of sickness benefits and missed income tax. Having a person return to work sooner would decrease the costs and increase the amount of available resources. However, to only have societal contribution as grounds for priority would mean that all health care would be directed towards the productive part of the population and not to those with the greatest need, who despite care cannot contribute to society. At the same time, in order to have resources for those with the greatest need there needs to be a substantial amount of resources in the first place. Ignoring the broader cost-benefit principle in circumstances where such resources are very scarce would mean that fewer of those with great needs would get any care.

One final question relates to the tensions between the forensic and therapeutic roles of health care professionals. Health care ethics stress the need for voluntary consent while the social insurance agency demands that patients must accept interventions that promote their work ability. However, treatments come with side-effects and the question then arises of to what extent a patient should accept side-effects of a treatment under the threat of losing their sickness benefits if they refuse. Should the social insurance agency refuse sickness benefits to patients who prefer symptom reduction over increase in work ability?

In this paper we have investigated the ethical frameworks of three key actors in the process of facilitating return to work among employees on sick leave: the employer, the social insurance agency and the health care system. In conclusion, our analysis indicates that potential tensions exist between the key actors in the return-to-work process and that these tensions need to be addressed in order to enable an increased cooperation between the actors, facilitating the development of a feasible plan of action for all parties, including the employee.

## Footnotes

1 We realise that the term common mental disorder may not be the preferred term by everyone. However, it is the standard term in the field and common mental disorders are the driving force behind policy change and the object of many targeted interventions [1–4]. In some contexts, the term AMI – any mental illness – is used, however this term is too broad for the present paper as it also encompasses severe mental disorders.

2 Swedish law does not require mental capacity for treatment consent or refusal unless the decision concerns life-sustaining treatment.

3 That is not to say that a person with mental disorder or even that a person who lacks mental capacity cannot hold a job. However, if the person is on sick-leave due to mental disorder then by definition that mental disorder makes them unable to do their job.
